# Factors Associated with Streptococcal Bacteremia in Diarrheal Children under Five Years of Age and Their Outcome in an Urban Hospital in Bangladesh

**DOI:** 10.1371/journal.pone.0154777

**Published:** 2016-05-02

**Authors:** Abu Sadat Mohammad Sayeem Bin Shahid, Tahmeed Ahmed, K. M. Shahunja, Senjuti Kabir, Fahmida Chowdhury, Abu Syeed Golam Faruque, Sumon Kumar Das, Mohammad Habibur Rahman Sarker, Pradip Kumar Bardhan, Mohammod Jobayer Chisti

**Affiliations:** 1 Infectious Diseases Division, International Centre for Diarrhoeal Disease Research, Bangladesh (icddr,b), Dhaka, Bangladesh; 2 Nutrition & Clinical Services Division, International Centre for Diarrhoeal Disease Research, Bangladesh (icddr,b), Dhaka, Bangladesh; East Carolina University Brody School of Medicine, UNITED STATES

## Abstract

**Background:**

Although Streptococcal bacteremia is common in diarrheal children with high morbidity and mortality, no systematic data are available on Streptococcal bacteremia in diarrheal children. We sought to evaluate the factors associated with Streptococcal bacteremia in diarrheal children under five years of age and their outcome.

**Methods:**

We used an unmatched case-control design to investigate the associated factors with Streptococcal bacteremia in all the diarrheal children under five years of age through electronic medical record system of Dhaka hospital of International Centre for Diarrhoeal Disease Research, Bangladesh. We had simultaneously used a retrospective cohort design to further evaluate the outcome of our study children. All the enrolled children had their blood culture done between January 2010 and December 2012. Comparison was made among the children with (cases = 26) and without Streptococcal bacteremia (controls = 78). Controls were selected randomly from hospitalized diarrheal children under five years of age.

**Results:**

Cases had proportionately higher deaths compared to controls, but it was statistically insignificant (15% vs. 10%, p = 0.49). The cases more often presented with severe dehydration, fever, respiratory distress, severe sepsis, and abnormal mental status compared to the controls (for all p<0.05). In the logistic regression analysis, after adjusting for potential confounders, it has been found that Streptococcal bacteremia in diarrheal children under five years of age was independently associated with nutritional edema (OR: 5.86, 95% CI = 1.28–26.80), hypoxemia (OR: 19.39, 95% CI = 2.14–175.91), fever (OR: 4.44, 95% CI = 1.13–17.42), delayed capillary refill time (OR: 7.00, 95% CI = 1.36–35.93), and respiratory distress (OR: 2.69, 95% CI = 1.02–7.12).

**Conclusions and Significance:**

The results of our analyses suggest that diarrheal children under five years of age presenting with nutritional edema, hypoxemia, fever, delayed capillary refill time, and respiratory distress may be at risk of Streptococcal bacteremia. It underscores the importance of identification of these simple clinical parameters for the prompt recognition and management in order to reduce the morbidity and death of such children especially in resource limited settings.

## Introduction

Bacteremia is one of the leading causes of childhood morbidity and mortality both in developed and developing countries [1, 2]. Blood cultures remain the “gold standard” test for detection of bacteremia. *Streptococcus* spp. are Gram-positive cocci, structurally encapsulated, and responsible for a variety of diseases in humans. It is one of the leading causes of pneumonia, meningitis, and bacteremia in all age groups particularly in younger children and adults [3, 4]. The organism commonly causes bacterial meningitis, especially in younger age group in developing countries [5]. Streptococcal infection generally presents in extreme ages with soft tissue infections, cardiac, renal, and splenic dysfunctions. There is estimated to be as high as 2 million deaths globally in adults each year due to streptococcal infections [3, 6–8]. Following effective introduction of pneumococcal conjugate vaccine, incidence of invasive streptococcal diseases was reduced in children in developed as well as developing countries [9–11]. The global burden of streptococcal disease is under-appreciated. It is estimated to cause 1.6–2.2 million deaths in children every year worldwide, mostly in developing countries. Among them 0.7–1 million are children under five years of age. Most of the deaths are associated with meningitis or pneumonia [5, 9, 10, 12]. Fatality in young children for septicemia and meningitis were 20% and 50%, respectively, in developing countries [13].

Although, mortality in children with Streptococcal bacteremia who had received WHO recommended antibiotics was still high [14], the virulence of streptococcal infection in diarrheal children is not well understood. *Streptococcus* has been found to have higher resistance to cotrimoxazole, ciprofloxacin, and erythromycin and lower resistance to penicillin in a number of studies involving children with pneumonia and/or sepsis [15–19]. Mortality may be even higher when children with Streptococcal bacteremia also suffer from diarrhea, although Gram-negative bacteremia is predominate among diarrheal children, as observed in previous studies in the last few decades [20–22]. Breaches of integrity of bowel mucosa followed by reduced immunity are perceived to be common in diarrheal children that make them more vulnerable to bacteremia. Consequently, clinical features suggestive of sepsis [23] in diarrheal children with Streptococcal bacteremia are believed to be more fatal compared to those without diarrhea. However, we do not have any published data to support this speculation. Thus, children with Streptococcal bacteremia and diarrhea need careful attention.

An understanding of the factors associated with Streptococcal bacteremia in diarrheal children may help clinicians in identifying and managing such children promptly and efficiently in order to reduce morbidity and mortality, especially in resource-poor settings. However, there are no published data on the factors that may predict Streptococcal bacteremia in children with diarrhea. Thus, the aim of our study was to evaluate the factors associated with Streptococcal bacteremia in diarrheal children under five years of age and their outcome.

## Materials and Methods

### Ethics Statement

The data used in this study was retrieved from case records of patients of Dhaka hospital of International Centre for Diarrhoeal Disease Research, Bangladesh (icddr,b). Data was entered in an anonymized and de-identified manner prior to analysis and was used for the improvement of the quality of care of the hospital patients. The study did not involve any interviews with patients or their care givers. No authors had any access to patient-identifying information. Waiver of an ethical approval from the institutional review board (the name of the review board of icddr,b is ‘Ethical Review Committee’) for publication was taken.

### Study Population and Site

We conducted our study in Dhaka hospital of International Centre for Diarrhoeal Disease Research, Bangladesh (icddr,b). The description of the study site has recently been published [24].

### Study Design

For this study, we used an unmatched case-control design to evaluate the factors associated with Streptococcal bacteremia in diarrheal children under five years of age and simultaneously a retrospective cohort design to assess the outcome among the groups. Data on diarrheal children of either sex, aged 0–59 months, admitted to the inpatient ward of Dhaka hospital of icddr,b from January 2010 to December 2012, who had a blood culture done because of suspected clinical sepsis [23], were extracted from SHEBA, an online database of Dhaka hospital of icddr,b. Children under five years of age with Streptococcal (comprising of *Streptococcus pneumoniae* and *Streptococcus* species) bacteremia constituted the cases, and those without Streptococcal bacteremia constituted the controls. Controls were randomly selected from the children without Streptococcal bacteremia by computer randomization using SPSS (version 17.0; SPSS Inc., Chicago) from the personal computerized data source of this study. A 1:3 unmatched case-control ratio was used to increase the statistical power of our analyses.

### Measurements

Case report forms (CRF) were developed, pretested, and finalized for data acquisition. Characteristics analyzed include demographics (age, gender, and type of residence and drinking water), fever (axillary temperature ≥38°C), cough, history of respiratory distress, convulsion, hypoxemia (defined as SpO_2_<90% as study site is at sea level [14], acute watery diarrhea (defined as the passage of three or more abnormally loose or watery stool in the previous 24 hours) [25], dehydration status, severe wasting (z score for weight for length/height <-3 of the WHO growth standard), severely underweight (z score for weight for age <-3 of the WHO growth standard), abnormal mental status (disorientation or lethargy or irritable) on admission, and outcome of the study children.

### Analysis

All the data were entered into SPSS for Windows (version 17.0; SPSS Inc., Chicago) and Epi-Info (version 6.0, USD, Stone Mountain, GA). In qualitative variables, differences in proportions were compared by the Chi-square test. In normally distributed quantitative data, differences of mean were compared by the Student's *t*-test; the Mann-Whitney test was used for comparison of data that were not normally distributed. A probability of less than 0.05 was considered statistically significant. Strength of association was determined by calculating the odds ratio (OR) and their 95% confidence intervals (CIs). In the uni-variate model (2/2 table), characteristics that were analyzed include age, sex, severe dehydration, fever, respiratory distress, nutritional edema, abnormal mental status, severe sepsis, hypoxemia, duration of vomiting, and delayed capillary refill time. Finally logistic regression analysis was performed to identify characteristics that were independently associated with Streptococcal bacteremia after adjusting for potential confounders. All categorical variables significantly associated with Streptococcal bacteremia in uni-variate analyses were included in the model as independent variables where Streptococcal bacteremia was the dependent variable.

## Results

**Fig 1:**
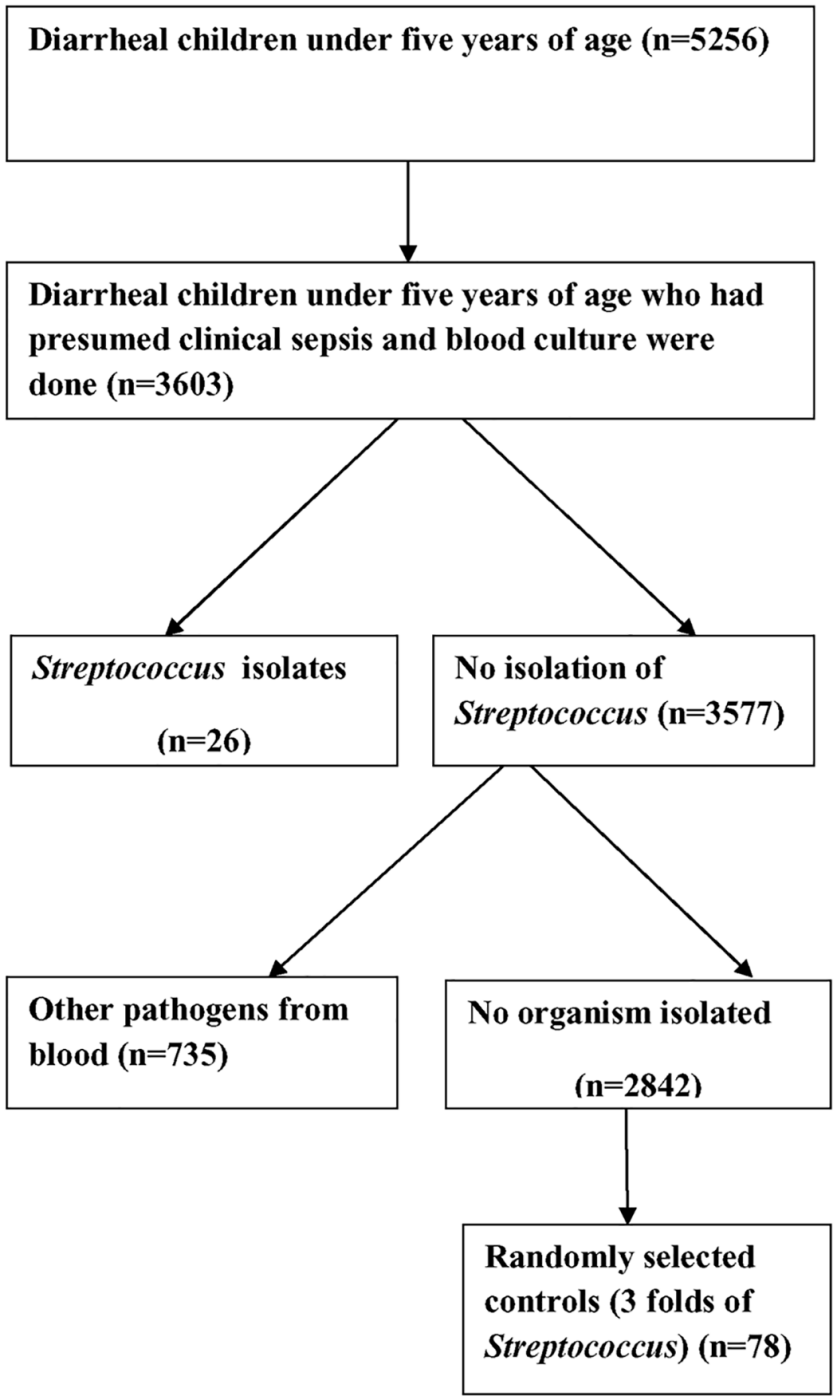
Flow-Chart of diarrheal children under five years of age who had *Streptococcus* species isolated from their blood and selection of their controls.

During the study period, the total number of diarrheal children under five years of age admitted was 5256, from whom 3603 children had presumed sepsis and their blood culture was performed and cases and controls were drawn from that (Fig 1). Among the cases (n = 26), there were 16 children who had *Streptococcus pneumoniae* and 10 who had *Streptococcus* species in their blood culture. Male:female ratio among cases and controls were 1.2:1 and 1.3:1, respectively. Characteristics of the diarrheal children with Streptococcal bacteremia that includes median duration of symptoms, such as diarrhea, vomiting, cough, fever, and respiratory distress, have been provided in Table 1. Median age of the patients among cases was 6.5 months and that of controls was 8 months. Serum creatinine level which was routinely done to evaluate the kidney function of the cases and the controls were 52 μmol/L and 33μmol/L, respectively, and the difference was not statistically significant (Table 2).

**Table 1:** Characteristics of diarrheal children under five years of age with (cases) or without (controls) Streptococcal bacteremia on presentation at hospital.

Characteristics	Cases (n = 26)	Controls (n = 78)
Female	12 (46)	34 (44)
Age in months (Median, IQR)	6.5 (4.75, 13.0)	8.0 (5.0, 12.0)
Stopped breast feeding before 6 months of age	9 (35)	18 (23)
Not immunization as per EPI schedule	1 (4)	7 (9)
Antibiotics given prior to admission	1 (4)	10 (13)
Received inotrope during hospitalization	3 (11)	7 (9)
Received blood transfusion during hospitalization	3 (11)	4 (5)
Acute watery diarrhea on admission	21 (81)	59 (76)
Duration of diarrhea in days (Median, IQR)	3.0 (1.0, 6.0)	4.0 (3.0, 7.0)
Presence of severe dehydration on admission	5 (19)	4 (5)
Presence of vomiting on admission	22 (85)	62 (79)
Duration of vomiting in days (Median, IQR)	2.5 (1.0, 4.25)	4.0 (2.0, 5.0)
Presence of fever on admission	23 (89)	51 (65)
Duration of fever in days (Median, IQR)	2.0 (1.0, 3.0)	3.0 (2.0, 5.0)
Presence of cough on admission	18 (69)	37 (47)
Duration of cough in days (Median, IQR)	3.0 (2.0, 5.0)	3.0 (2.0, 5.0)
Presence of respiratory distress on admission	16 (62)	27 (35)
Duration of respiratory distress in days (Median, IQR)	2.0 (1.0, 2.0)	2.0 (2.0, 3.0)
Presence of edema on admission	6 (23)	4 (5)
Presence of convulsion on or after admission	7 (27)	11 (14)
Presence of lower chest wall in-drawing on admission	16 (62)	27 (35)
Presence of severe sepsis on or after admission	3 (12)	1 (1)
Presence of urinary tract infection on admission	5 (19)	25 (32)
Presence of hypoglycemia on or after admission	1 (4)	5 (6)
Presence of hypoxemia (SPO_2_< 90%) on admission	6 (23)	1 (1)
Abnormal mental status on admission	16 (62)	27 (35)
Severe wasting (Weight for length/height z score <-3)	9 (43)	25 (32)
Severely underweight (Weight for age z score <-3)	14 (54)	49 (63)
Presence of delayed capillary refill time (≥3 seconds)	8/14(57)	4/18(22)
Duration of hospital stay in days (Median, IQR)	6.0 (4.0, 13.25)	6.0 (4.0, 10.0)
Total WBC count (number/cumm) (Median, IQR)	18415 (11000, 28990)	14000 (10000, 20700)
Serum creatinine level (μmol/L) (Median, IQR)	52.0 (22.0, 76.8)	32.7 (19.7, 62.9)

n: number; Parenthesis at the right side of the dichotomous variable denotes ‘%’ until specified otherwise; IQR: Inter-quartile range;

**Table 2:** Comparison of characteristics of diarrheal children under five years of age with (cases) or without (controls) Streptococcal bacteremia at hospitalization and their outcome during hospitalization.

Characteristics	Cases (n = 26)	Controls (n = 78)	OR	95% CI
*On presentation*				
Female	12 (46)	34 (44)	1.11	0.41–2.96
Age in months (Median, IQR)	6.5 (4.75, 13.0)	8.0 (5.0, 12.0)	1.01	0.96–1.05
Stopped breast feeding before 6 months of age	9 (35)	18 (23)	1.76	0.60–5.12
Not immunization as per EPI schedule	1 (4)	7 (9)	0.41	0.02–3.60
Antibiotics given prior to admission	1 (4)	10 (13)	0.26	0.01–2.14
Received inotrope during hospitalization	3 (11)	7 (9)	1.32	0.25–6.39
Received blood transfusion during hospitalization	3 (11)	4 (5)	2.41	0.39–14.15
Acute watery diarrhea on admission	21 (81)	59 (76)	1.35	0.41–4.75
Duration of diarrhea in days (Median, IQR)	3.0 (1.0, 6.0)	4.0 (3.0, 7.0)	1.04	0.95–1.15
Presence of severe dehydration on admission	5 (19)	4 (5)	4.40	0.91–21.96
Presence of vomiting on admission	22 (85)	62 (79)	1.42	0.39–5.66
Duration of vomiting in days (Median, IQR)	2.5 (1.0, 4.25)	4.0 (2.0, 5.0)	1.30	0.98–1.71
Presence of fever on admission	23 (89)	51 (65)	4.06	1.02–18.72
Duration of fever in days (Median, IQR)	2.0 (1.0, 3.0)	3.0 (2.0, 5.0)	1.26	0.97–1.63
Presence of cough on admission	18 (69)	37 (47)	2.49	0.89–7.15
Duration of cough in days (Median, IQR)	3.0 (2.0, 5.0)	3.0 (2.0, 5.0)	1.14	0.87–1.51
Presence of respiratory distress on admission	16 (62)	27 (35)	3.02	1.10–8.39
Duration of respiratory distress in days (Median, IQR)	2.0 (1.0, 2.0)	2.0 (2.0, 3.0)	1.27	0.85–1.91
Presence of edema on admission	6 (23)	4 (5)	5.55	1.22–26.50
Presence of convulsion on or after admission	7 (27)	11 (14)	2.24	0.67–7.43
Presence of lower chest wall in-drawing on admission	16 (62)	27 (35)	3.02	1.10–8.39
Presence of severe sepsis on or after admission	3 (12)	1 (1)	10.04	0.86–263.7
Presence of urinary tract infection on admission	5 (19)	25 (32)	0.46	0.10–2.00
Presence of hypoglycemia on or after admission	1 (4)	5 (6)	0.23	0.01–2.37
Presence of hypoxemia (SPO_2_< 90%) on admission	6 (23)	1 (1)	23.10	2.49–539.5
Abnormal mental status on admission	16 (62)	27 (35)	3.02	1.10–8.39
Severe wasting (Weight for length/height z score <-3)	9 (43)	25 (32)	1.59	0.53–4.74
Severely underweight (Weight for age z score <-3)	14 (54)	49 (63)	0.69	0.26–1.86
Presence of delayed capillary refill time (≥3 seconds)	8/14(57)	4/18(22)	4.67	1.01–21.65
Serum creatinine level (μmol/L) (Median, IQR)	52.0 (22.0, 76.8)	32.7 (19.7, 62.9)	0.99	0.98–1.01
*Outcome during hospitalization*				
Deaths	4 (15)	8 (10)	1.59	0.36–6.65
Recovery	20 (77)	66 (85)	0.60	0.20–1.82
Others(Referred/Left against medical advice)	2 (7)	4(5)	1.54	0.27–8.95

n: number; parenthesis at the right side of the dichotomous variable denotes ‘%’ until specified otherwise; OR: Odds ratio; CI: Confidence interval; IQR: Inter-quartile range;

The cases had proportionately higher death rates compared to the controls (15% vs. 10%) but it was statistically insignificant (p = 0.49) (Table 2). Among the cases and the controls, 20 (77%) and 66 (85%) were discharged following recovery from acute illnesses and 2 (7%) and 4 (5%) were referred and left the hospital, respectively (Table 2). In uni-variate analysis, the cases more often presented with severe dehydration, fever, respiratory distress, severe sepsis, abnormal mental status, and delayed capillary refill time (seconds) compared to the controls (Table 2). Other variables in Table 2 were comparable among the groups. In the logistic regression analysis, after adjusting for potential confounders, it has been found that Streptococcal bacteremia had the independent association with nutritional edema, hypoxemia, fever, respiratory distress, and delayed capillary refill time (Table 3). There was higher sensitivity of penicillin (92%), ampicillin (94%), and ceftriaxone (100%) and lower sensitivity of cotrimoxazole (12.5%), ciprofloxacin (33%), and erythromycin (61%) in *Streptococcus* isolates from these patients. The sensitivity pattern of these *Streptococcus* isolates to other antibiotics in Table 4.

**Table 3:** Results of logistic regression to explore the independent association of Streptococcal bacteremia in diarrheal children under five years of age.

Characteristics	Adjusted OR	95% CI	p
Hypoxemia	19.39	2.14–175.91	<0.01
Edema	5.86	1.28–26.80	0.02
Fever	4.44	1.13–17.42	0.03
Respiratory distress	2.69	1.02–7.12	0.04
Delayed capillary refill time	7.00	1.36–35.93	0.02

Hypoxemia was adjusted for severe sepsis, severe dehydration, and abnormal mental status; Nutritional edema was adjusted for fever, severe sepsis, and respiratory distress; Fever was adjusted for severe sepsis, respiratory distress, and nutritional edema; Respiratory distress was adjusted for severe sepsis, fever and nutritional edema; Delayed capillary refill time was adjusted for duration of vomiting, abnormal mental status, and severe dehydration;

**Table 4:** Antibiotic sensitivity for the Streptococcal isolates in blood (n = 26).

Name of antibiotic	Sensitive to antibiotic (% sensitivity)
Ampicillin	17/18 (94)
Amoxycillin	6/6 (100)
Chloramphenicol	1/1 (100)
Cotrimoxazole	2/16 (12.5)
Ciprofloxacin	1/3 (33)
Penicillin G	23/25 (92)
Ceftriaxone	25/25 (100)
Cefixime	17/24 (71)
Levofloxacin	11/12 (92)
Ceftazidime	1/1 (100)
Azythromycin	12/15 (80)
Erythromycin	14/23 (61)
Cephradine	1/1 (100)
Gentamicin	1/12 (8)
Oxacillin	7/8 (87)
Vancomycin	2/2 (100)

## Discussion

The main observation of this chart review is the association of nutritional edema and hypoxemia with Streptococcal bacteremia. Nutritional edema is often responsible for depressed cell mediated and humoral immune responses, frequently associated with the impairment of IgA production, chemotaxis, reduced mature T cells, and compromised phagocytic activity [26, 27]. As a result, children with nutritional edema become highly susceptible to rapidly deteriorating infectious bacteremia, [28] such as Streptococcal bacteremia. Streptococcal bacteremia often leads to vasodilation and capillary leakage, resulting in amplified cytokines or other inflammatory stimuli which leads to poor peripheral microcirculation and hypotension [29, 30]. This phenomenon is associated with mixed metabolic and respiratory acidosis leading to severe respiratory distress [31]. This respiratory distress results in impairment of alveolar arterial oxygen diffusion and a concomitant increase in the partial pressure of carbon-dioxide (CO_2_) due to abnormally lower alveolar ventilation [32], ultimately resulting in hypoxemia [33].

The observation of the lack of an association of severe dehydration, severe sepsis, duration of vomiting and abnormal mental status with Streptococcal bacteremia in logistic regression analysis after adjusting for potential confounders (Table 3) underscores the importance of the true association of nutritional edema, hypoxemia, fever, delayed capillary refill time, and respiratory distress with Streptococcal bacteremia. However, a number of previous studies from developing countries, including two from Bangladesh, showed a significant association of dehydrating diarrhea, severe sepsis, and abnormal mental status with bacteremia [20, 34–36].

Fever, delayed capillary refill time, and respiratory distress were also independently associated with Streptococcal bacteremia in our study children. Fever and delayed capillary refill time are essential components of inflammatory responses, especially in Streptococcal bacteremia [35, 37], and respiratory distress is often found in metabolic acidosis as a ramification of bacteremia in children [31].

Physicians in healthcare settings in developing countries like Bangladesh mostly rely on their clinical judgment for determining the need for antimicrobial therapy. Our chart review underscores the importance of the use of clinical parameters such as identified nutritional edema, hypoxemia, fever, delayed capillary refill time, and respiratory distress in treating sick children prone to have Streptococcal bacteremia. This will minimize the delay in initiating the treatment with aggressive antibiotics without delaying for blood culture results. However, the wide availability of pulse oximeter in resource limited settings is imperative for the routine measurement of hypoxemia.

The next important observation of our chart review is the proportionately higher death rates in diarrheal children under five years of age with Streptococcal bacteremia compared to those without Streptococcal bacteremia; although, this is statistically insignificant. However, a number of previous studies had found the association of higher case-fatality rates with Streptococcal bacteremia [38, 39]. The failure of attaining the significant difference in deaths in our study might be due to selection of sick controls as they all had diarrhea and/or other co-morbidities such as edematous malnutrition and severe sepsis. Lower number of cases might also have an impact on statistical insignificance. However, bacteremia appears to be an important link between diarrheal illness and death in Bangladeshi children in previous studies [40].

Our observation of a higher sensitivity of penicillin, ampicillin, and ceftriaxone and a lower sensitivity of cotrimoxazole, ciprofloxacin, and erythromycin in streptococcal isolates in blood is very important. This underscores the importance of the use of ampicillin as one of the drugs of choice in childhood sepsis as advocated by WHO. Another drug of first line therapy of childhood sepsis recommended by WHO is gentamicin, and it is believed to treat potential Gram-negative bacterial infections. Recent data from Bangladesh are consistent with this observation [41]. Our data also justifies the recommendation of ceftriaxone as second line therapy of childhood sepsis by WHO. However, poor sensitivity of cotrimoxazole, ciprofloxacin, and erythromycin in streptococcal isolates is alarming and might be due the fact that these drugs are illegally sold by drug sellers in Bangladesh without any prescription by qualified physicians. Thus, clinicians in developing countries should follow the WHO recommendation for the management of childhood sepsis or suspected Streptococcal bacteremia in children.

We failed to observe any significant difference in age, gender, presence of acute watery diarrhea on admission, presence of cough on admission, severe wasting, severely underweight, and serum creatinine level between the groups. This might be due to the smaller sample size. However, failure to identify any statistical difference in these parameters among the groups might be due to the fact that there was a lack of documentation in the digital record system (SHEBA).

Our study has potentially four important limitations. First, the retrospective nature of the study in identifying Streptococcal bacteremia prevented us from collecting information on a broader range of variables that may have been potential additional factors. Second, the limited number of patients included in this study might have reduced the power of the analyses and lessened the ability to identify more subtle differences between the groups and identify further relevant factors associated with Streptococcal bacteremia. Third, lack of selection of healthy controls limited our ability to identify true risk factors for Streptococcal bacteremia. Fourth, the numbers of tested *Streptococcus* isolates for sensitivity of different antibiotics by our reference laboratory of icddr,b were very small which further limited the generalisibility of the sensitivity pattern of antibiotics to *Streptococcus*.

In conclusion, the results of our data analyses suggest that diarrheal children under five years of age presenting with nutritional edema, hypoxemia, fever, respiratory distress, and delayed capillary refill time are associated with Streptococcal bacteremia. Ampicillin and Ceftriaxone are found to have higher sensitivity to streptococcal isolates in blood. This underscores the importance of prompt identification of these simple clinical parameters and simultaneously treating these children with WHO recommended antibiotics that may help to reduce morbidity and mortality of such children especially in resource-poor settings. However, a further study with a larger sample and a prospective design is imperative to accept or refute our observation.
